# Acute Myocardial Infarction (AMI) as the Effect Modifiers to Modify the Association Between Red Blood Cell Distribution Width (RDW) and Mortality in Critically Ill Patients With Stroke

**DOI:** 10.3389/fmed.2022.754979

**Published:** 2022-04-26

**Authors:** Tongli Guo, Zuoan Qin, Dian He

**Affiliations:** ^1^Department of Neurology, Affiliated Hospital of Guizhou Medical University, Guiyang, China; ^2^Department of Cardiology, The First People's Hospital of Changde City, Changde, China

**Keywords:** red blood cell distribution width, acute stroke, mortality, critically ill, intensive care unit (ICU), eICU Collaborative Research Database

## Abstract

**Background and Objectives:**

Few studies have evaluated the impact of red blood cell distribution width (RDW) on prognosis for critically ill patients with acute stroke according to recent studies. The aim of this study was to investigate the association between RDW and mortality in these patients.

**Methods:**

Clinical data were extracted from the eICU Collaborative Research Database (eICU-CRD) and analyzed. The exposure of interest was RDW measured at admission. The primary outcome was in-hospital mortality. Binary logistic regression models and interaction testing were performed to examine the RDW-mortality relationship and effect modification by acute myocardial infarction and hypertension (HP).

**Results:**

Data from 10,022 patients were analyzed. In binary logistic regression analysis, after adjusting for potential confounders, RDW was found to be independently associated with in-hospital mortality {odds ratio (OR) 1.07, [95% confidence interval (CI) 1.03 to 1.11]; *p* = 0.001}. Higher RDW linked to an increase in mortality (OR, 1.07; 95% CI, 1.03 to 1.11; P for trend < 0.0001). Subgroup analysis showed that, in patients combined with AMI and without HP (both P-interaction <0.05), the correlation between RDW and in-hospital mortality is stronger (AMI group: OR, 1.30; 95% CI, 1.07 to 1.58, not the AMI group: OR, 1.06; 95% CI, 1.02, 1.10; the HP group: OR,.98; 95% CI,.91 to 1.07, not the HP group: OR, 1.09; 95% CI, 1.05 to 1.14).

**Conclusions:**

A higher baseline RDW is independently correlated with prognosis in critically ill patients with acute stroke, and the correlation can be modified by AMI and HP duration.

## Introduction

The RDW is a simple, rapid, and easily available assessment measure of erythrocyte volume variability and heterogeneity (1). Recently, besides hematological disorders, RDW has emerged as a potential biomarker in many critical clinical cases, mainly in cardiovascular and cerebrovascular diseases (2, 3).

Globally, the aging of the population and the accumulation of risk factors lead to an increased lifetime risk of stroke (4). According to a report from the American Heart Association, the mortality rate after stroke was 10.5, 21.2, 39.8, and 58.4% at 30 days after stroke, 1 year after stroke, and 5 years after stroke or even longer, respectively (5, 6). Given complicated pathophysiological mechanisms of stroke, outcome prognostication in patients with stroke remains challenging. The ability of existing stroke prognostic prediction models was limited. Blood-based biomarkers might provide additional information to established prognostic factors (7, 8).

As far as existing research was concerned, no uniform conclusions had been drawn thus far about the role of RDW in the prognosis of stroke (9). There were several studies investigating the connection between RDW and stroke. A meta-analysis including a total of 31 studies with 3,487,896 patients concluded that higher baseline RDW could be as a predictor of stroke occurrence and an unfavorable functional outcome; higher mortality was found in patients with increased RDW (OR/RR, 1.278; 95% CI = 1.221,1.337) (10). Zhao et al. (11) analyzed 4,314 patients with stroke, retrospectively, and the results showed that the hazard ratio (95%, CI) of mortality for the second (RDW: 13.4–14.3%) and third (>14.3%) tertiles was 1.15 (0.96, 1.37) and 1.40 (1.17, 1.68), respectively, compared with the reference group (RDW <13.4%). However, Ntaios et al. (12) retrospectively analyzed 1,504 patients with stroke with multivariate robust regression analysis, and the findings manifested that RDW was significantly associated with a functional outcome (OR = 10.73 for a poor outcome, *p* < 0.001) at univariate analysis but not multivariate. The clinical significance of RDW in stroke has not been comprehensively investigated, owing to the differences in study population, study design, adjustment for covariates. Therefore, to provide additional evidence on the potential association of baseline RDW with prognosis following acute stroke, we performed a retrospective cohort study using the eICU Collaborative Research Database (eICU) (13), which is an available multi-center database, including 58 hospitals in the USA.

## Materials and Methods

### Study Design

This research concerns a *post hoc* analysis of data from eICU. The target independent variable is RDW. We obtained the information of RDW-CV at the baseline measured at admission and recorded as a continuous variable. The outcome variable is in-hospital mortality (Y = 1 survivor, Y = 0 non-survivor).

### Data Source

Data were collected from the eICU database, which comprises 200,859 ICU patients admitted to 208 hospitals located throughout the United States between 2014 and 2015 (14). All patients in the eICU databases were eligible for inclusion in the present investigation. The following patients were excluded: (1) patients who had been admitted to the ICU more than one time; (2) patients lacking of information on survival status; (3) RDW information missing. The use of the data had been approved by the Institutional Review Boards of Massachusetts Institute of Technology (Cambridge, MA, USA). After finishing the web-based training courses and the Protecting Human Research Participants examination (No. 36208651,), we obtained permission to extract data from eICU-CRD (14).

### Participants

Patients with a diagnosis of brain stroke recorded on the patient dataset were potentially eligible. The patients with stroke can be grouped into three categories: ischemic stroke group, hemorrhagic stroke group, and others group.

### Covariates

The covariates involving in this study were selected based on our clinical experiences and studies from other existing risk factors in mortality in the stroke cohort. Based on the principles above, accordingly, the following variables were used as covariates: (1) continuous variables: age; BMI. (2) categorical variables: gender; GCS group; ethnicity; stroke type; comorbidities on admission:acute respiratory failure, coagulopathy, diabetes mellitus, hypertension, sepsis, cancer, and AMI.

### Statistical Analysis

Continuous variables were presented as mean with standard deviations and categorical variables as total number and percentage. Due to sensitivity analysis, continuous variables were compared using theANOVA and Kruskal Wallis rank sum test. To evaluate the link between RDW and mortality in the stroke cohort, we constructed three distinct models using the binary logistic regression model, including the univariate logistic regression model (no covariates were adjusted), the minimally adjusted model (only sociodemographic variables were adjusted), and the fully adjusted model (covariates presented in Table 1 were adjusted), and effect sizes with 95% confidence intervals were recorded. The adjustment strategy for the subgroup analyses was the same as the fully adjusted model, except for the stratification factor, which was not adjusted; all other factors were adjusted. The interaction of subgroups testing was performed using likelihood ratio tests. To test the robustness of our results, we performed a sensitivity analysis. We converted RDW into a categorical variable according to the quartile, and calculated the P for a trend in order to verify the results of RDW as the continuous variable, and to observe the possibility of non-linearity. Data were analyzed using the statistical software packages R (http://www.R-project.org, The R Foundation) and EmpowerStats (http://www.empowerstats.com/; X&Y Solutions, Inc, Boston, MA). All statistical tests were 2-sided, and a *P*-value <0.05 was considered statistically significant.

**Table 1:** Baseline characteristics of participants.

**RDW (quartile)**	**Q1**	**Q2**	**Q3**	**Q4**	***P*** **-value**	***P*** -value^*^
N	2206	2763	2460	2593		
RDW, mean + SD, %	12.69 ± 0.34	13.54 ± 0.23	14.44 ± 0.31	17.07 ± 2.17	<0.001	<0.001
Age, mean ± SD, year	62.47 ± 15.61	67.27 ± 14.45	69.47 ± 14.08	68.49 ± 14.48	<0.001	<0.001
BMI, mean + SD	27.10 ± 6.99	27.27 ± 7.81	27.64 ± 8.03	27.33 ± 8.62	0.132	<0.040
**GCS group**, ***n*** **(%)**					<0.001	-
15 (normal)	1269 (58.70%)	1402 (51.98%)	1102 (45.94%)	1011 (40.46%)		
13–14 (mild)	372 (17.21%)	487 (18.06%)	477 (19.88%)	523 (20.93%)		
9–12 (morderate)	252 (11.66%)	429 (15.91%)	411 (17.13%)	486 (19.45%)		
3–8 (severe)	269 (12.44%)	379 (14.05%)	409 (17.05%)	479 (19.17%)		
**Gender**, ***n*** **(%)**					<0.001	-
Male	1225 (55.53%)	1456 (52.72%)	1285 (52.24%)	1230 (47.44%)		
Female	981 (44.47%)	1306 (47.28%)	1175 (47.76%)	1363 (52.56%)		
**Ethniciy**, ***n*** **(%)**					<0.001	-
African American	159 (7.25%)	225 (8.19%)	336 (13.71%)	493 (19.12%)		
Asian	72 (3.28%)	63 (2.29%)	47 (1.92%)	47 (1.82%)		
Caucasian	1732 (78.98%)	2145 (78.11%)	1840 (75.07%)	1798 (69.72%)		
Hispanic	88 (4.01%)	109 (3.97%)	85 (3.47%)	119 (4.61%)		
Native American	4 (0.18%)	9 (0.33%)	16 (0.65%)	17 (0.66%)		
Unknown	138 (6.29%)	195 (7.10%)	127 (5.18%)	105 (4.07%)		
**Status at hospital discharge**, ***n*** **(%)**					<0.001	-
Alive	1982 (91.04%)	2402 (87.95%)	2086 (85.91%)	2028 (79.34%)		
Expired	195 (8.96%)	329 (12.05%)	342 (14.09%)	528 (20.66%)		
**Acute respiratory failure**, ***n*** **(%)**	255 (11.56%)	378 (13.68%)	441 (17.93%)	580 (22.37%)	<0.001	-
**Coagulopathy**, ***n*** **(%)**	32 (1.45%)	81 (2.93%)	94 (3.82%)	157 (6.05%)	<0.001	-
**Diabetes mellitus**, ***n*** **(%)**	216 (9.79%)	294 (10.64%)	377 (15.33%)	433 (16.70%)	<0.001	-
**Acute myocardial infarction**, ***n*** **(%)**	25 (1.13%)	77 (2.79%)	74 (3.01%)	78 (3.01%)	<0.001	-
**Hypertention**, ***n*** **(%)**	634 (28.74%)	883 (31.96%)	836 (33.98%)	784 (30.24%)	<0.001	-
**Sepsis**, ***n*** **(%)**	53 (2.40%)	117 (4.23%)	129 (5.24%)	304 (11.72%)	<0.001	-
**Stroke type**, ***n*** **(%)**					0.367	-
Hemorrhagic stroke	692 (46.95%)	885 (48.28%)	788 (47.21%)	794 (45.87%)		
Ischemic stroke	761 (51.63%)	930 (50.74%)	868 (52.01%)	912 (52.69%)		
Others	21 (1.42%)	18 (0.98%)	13 (0.78%)	25 (1.44%)		
**Cancer**, ***n*** **(%)**	13 (0.59%)	16 (0.58%)	12 (0.49%)	44 (1.70%)	<0.001	-

*Data are presented as mean ± stantard deviation and n (%). RDW, red blood distribution width; BMI, body mass index; GCS, Glasgow Coma Scale*.

* *P-value indicated ANOVA; P-value indicated Kruskal Wallis rank sum test. Q1 means RDW 12.69 ± 0.34, Q2 means RDW 13.54 ± 0.23, Q3 means RDW 14.44 ± 0.31, Q4 means RDW 17.07 ± 2.17*.

## Results

### Baseline Characteristics of Participants

There are a total of 200,859 patients, of which 189,752 are non-stroke patients. Among the remaining 11,107 people, no one lacks survival status information, and 1,085 lack RDW information. In the end, the remaining 10,022 patients were used for final data analysis. We presented baseline demographics and clinical characteristics of included 10,022 participants in Table 1. The population at the baseline, of whom 51.85% were male and 48.15% were female with a mean age of 67.09 ± 14.89 years. The mean RDW of study cases was 14.49% ± 1.99%. The mean duration of hospital stay in this cohort was 3.32 h, the median was 2.02 h, and the incidence of death was 13.91% (1,394/10,022). All the patients were divided into quartiles on the basis of baseline RDW. Among them, there were 3,159 and 3,471 patients with ischemic stroke and hemorrhagic stroke, respectively. Compared with the RDW Q1 group (RDW, 11.40 to 13.10). Patients with a higher RDW were remarkably more likely to be older, female, and American to have reported comorbidities such as acute respiratory failure, AMI, diabetes mellitus, sepsis, coagulopathy, hypertension, and cancer (all *p* < 0.001). In contrast, there was little difference between the four groups in terms of stroke type (*p* = 0.367) and BMI (*p* = 0.132).

### Relationship Between RDW and In-Hospital Mortality in Patients With Stroke

The univariate logistic regression model showed that the increased RDW was associated with higher risk of death; the minimally adjusted model showed the same results. When we adjusted for gender, age, ethniciy, BMI, GCS group, acute respiratory failure, coagulopathy, diabetes mellitus, hypertension; sepsis, stroke type, cancer, and AMI, the OR (95% CI) of in-hospital mortality was 1.07 (1.03, 1.11, *p* = 0.001) in all included patients, which indicated that the increased 1 unit of RDW was associated with 7% increases of risk of in-hospital mortality. When RDW was categorized into four groups by RDW quartile levels, the OR (95% CI) of in-hospital mortality for the second (13.2–13.9%), third (14–15%), and fourth (15.1–32.2%) quartiles was 1.10 (0.85 to 1.43, *p* = 0.4635), 1.27 (0.98 to 1.64, *p* = 0.074) and 1.71 (1.33 to 2.19, *p* < 0.001), respectively. The results of P for a trend were significant (Table 2).

**Table 2:** Results of binary logistic regression model.

	**Non-adjusted model Effect size OR, 95%CI, *P*-value**	**Minimally-adjusted model** **Effect size OR, 95%CI, *P*-value**	**Fully-adjusted model Effect size OR, 95%CI, *P*-value**
RDW	1.14 (1.12, 1.17) <0.0001	1.14 (1.11, 1.17) <0.0001	1.07 (1.03, 1.11) 0.0010
**RDW (quartile)**			
Q1	Ref	Ref	Ref
Q2	1.39 (1.15, 1.68) 0.0005	1.30 (1.07, 1.57) 0.0068	1.10 (0.85, 1.43) 0.4635
Q3	1.67 (1.38, 2.01) <0.0001	1.54 (1.27, 1.86) <0.0001	1.27 (0.98, 1.64) 0.0740
Q4	2.65 (2.22, 3.15) <0.0001	2.50 (2.09, 2.99) <0.0001	1.71 (1.33, 2.19) <0.0001
P for trend	<0.0001	<0.0001	<0.0001

*Non-adjusted model: we do not adjusted for any covariates*.

*Minimally-adjusted model: only sex, age, and race are adjusted for*.

*Fully-adjusted model: all covariates presented in Table 1 are adjusted for*.

*Q1 means RDW 12.69 ± 0.34, Q2 means RDW 13.54 ± 0.23, Q3 means RDW 14.44 ± 0.31, Q4 means RDW 17.07 ± 2.17*.

*95%CI, 95% confidence interval*.

We also used interaction test to look for the potential modifying factors. As shown in Table 3 and Supplementary Table 1, gender, age, ethniciy, BMI, GCS group, acute respiratory failure, coagulopathy duration, diabetes mellitus duration, sepsis, stroke type, cancer duration were set as pre specified modifying factors. However, we only found that the comorbidities of AMI and HP can modify the correlation between RDW and in-hospital mortality as the Table 3 shows. In terms of AMI status, a stronger association between RDW and mortality can be detected in patients with AMI (1.30, 1.07 to 1.58) than that of in patients with non-AMI (1.06, 1.02 to 1.10), P for interaction is 0.0447. In terms of HP status, a stronger association between RDW and mortality can be detected in patients with non-HP (1.09, 1.05 to 1.14) than that of in patients with HP (0.98, 1.05 to 1.14), P for interaction is 0.0237. In addition, we did not find that the variables of age, BMI, gender, GCS, ethniciy, acute respiratory failure, coagulopathy duration, diabetes mellitus duration, sepsis, stroke type, and cancer duration can be modified the association between RDW and in-hospital mortality (all Ps for interaction are larger than 0.05), as the Supplementary Table 1 shows.

**Table 3:** Subgroup analysis by stratified binary logistic regression model.

**Parameters**	**OR**	**95%CI Low**	**95%CI High**	***P*** **-value**	**P (interaction)**
**Hypertention duration**					0.0237
No	1.09	1.05	1.14	<0.0001	
Yes	0.98	0.91	1.07	0.7006	
**Acute myocardial infarction**					0.0447
No	1.06	1.02	1.10	0.0040	
Yes	1.30	1.07	1.58	0.0091	

*95%CI, 95% confidence interval*.

## Discussion

The retrospective cohort study was designed to find the association of RDW with in-hospital mortality in patients with stroke based on eICU. Our results indicated that a higher RDW, in the short term, was significantly and positively connected with increased all-cause mortality in patients with stroke. In addition, our research showed that this would make patients with the complication of AMI face a greater risk of death compared with people without. A few previous cohort studies indicated that RDW was independently associated with an adverse functional outcome or death after stroke (8, 10, 15–20). Their findings were consistent with ours and provided support for our present study. A study of 847 consecutive patients with first-ever acute cerebral infarction within 7 days of the symptom onset reported that RDW was relevant to a poor functional outcome (modified Rankin Scale >2) and all-cause mortality at 3 months, and 1 year after the stroke onset (8). Cohort analysis of 274 adult patients with subarachnoid hemorrhage (SAH) admitted to ICU demonstrated that RDW was a useful index to evaluate hospital mortality and 1-year mortality (20). However, most of these studies were all limited by the relatively small sample size and did not include all stroke populations but only included ischemic stroke or SAH. Additionally, present reports had indicated that RDW is related to the prognosis of patients with AMI or acute coronary syndrome (21–25), but these studies had not clearly clarified the effect of the comorbidities on the relationship between RDW and death in patients with stroke. In contrast, there were two previous studies that had pointed out opposite results with ours as follows. A cohort study of 1,504 participants with ischemic stroke admitted in the Center Hospitalier Universitaire Vaudois (CHUV) Stroke Unit, Lausanne cleared that RDW did not predict a functional outcome. In the study, patients with intracerebral hemorrhage and SAH were excluded (11). Another population-based cohort study of 1,152 subjects found no association between RDW and risk of all-cause mortality among patients with incident stroke (the median follow-up time was 15.8 years) (26). It is worth noting that RDW was measured, on average, 7.4 years prior to the cerebrovascular event in the study; therefore, the timing between RDW measurement, stroke, and death could have influenced the findings. A retrospective cohort study of 180 patients who underwent CEA reported that increased RDW-CV was associated with increased long-term mortality (adjusted hazard ratio [aHR] 2.455, 95% CI, 1.231–4.894, *p* = 0.011) (27). A secondary analysis of the Medical Information Mart for Intensive Care III (MIMIC-III) database study showed RDW as an independent risk factor of all-cause mortality of patients with AKI (HR, 1.219, 95% CI, 1.211 to 1.228); RDW is positively correlated with survival time of a 4-year follow-up in critically ill patients with AKI (28). Therefore, we speculated that RDW could predict both short-term and long-term prognoses, and this speculation still needed more evidence to confirm.

There was no conclusive conclusion about the mechanism of RDW and increased mortality after stroke. The following were plausible explanations about the underlying biological mechanisms. A recent study has confirmed the independent association between RDW-standard deviation (RDW-SD) and serum neuron-specific enolase (NSE) levels, and NSE was used as a biomarker for evaluating neuronal damage and predicting the prognosis of stroke. These results indicated that RDW-SD is related to neuronal damage in patients with stroke (1). In the process of ischemic stroke (IS), the death of vascular endothelial cells destroyed the blood-brain barrier, leading to neuronal degeneration, swelling, and necrosis of glial cells. Subsequently, the NSE released by the injured neurons crosses the blood-brain barrier and rapidly diffused into the bloodstream through the ischemic tissue (29). Secondly, it had been found that RDW was positively correlated with plasma inflammatory biomarkers, such as C-reactive protein (CRP), erythrocyte sedimentation rate (ESR), and interleukin (IL)-6 (30–34). A study reported that inflammation was related to the process of IS, from initial ischemia to infarction and secondary repair (35, 36). Inflammation and oxidative stress may interfere with red blood cell production, inhibit red blood cell maturation, change the deformability of red blood cell membranes and the half-life of red blood cells in circulation, leading to an increase in RDW (37). Therefore, the elevated RDW increased the morbidity and mortality of IS by becoming a marker that increased inflammation, induced stroke, and led to a post-stroke state, or marked a prethrombotic state. Thirdly, higher RDW may reduce blood oxygen saturation, thereby reducing physiological reserves to resist hypoxic stress (38). In addition, it may cause abnormal blood coagulation and increase the risk of thromboembolism (39), and finally lead to stroke recurrence, which may exacerbate brain damage or lead to a worse prognosis. In view of the predictive role of RDW in many other diseases, RDW is more likely to be a general marker rather than a specific predictor of stroke (6). Based on the relationship between RDW and the prognosis of patients with stroke, future research should continue to explore whether the application of RDW can help doctors stratify the risk factors of stroke and secondary prevention in clinical practice to make clinical decisions more beneficial to patients.

This study had following strengths: Firstly, the sample size is large. Secondly, the interaction test expands our findings, that is, in patients with AMI and non-HP, an increase in RDW means a higher risk of death in patients, which will provide a better reminder for clinical decision-making. Thirdly, in this study, we ensured the robustness of the results through a series of sensitivity analyses (conversion of target independent variable form, subgroup analysis). This made our results more reliable.

Several limitations should be considered in the interpretation of our results. First, our study is limited to the American population; therefore, the conclusions of this study need to be cautious when applied to populations in other countries. Second, although we tried to adjust for possible confounding factors, However, residual confounders still cannot be ruled out completely. Finally, we did not exclude patients with hemoglobinopathies, which have been shown to increase RDW values like thalassemia and sickle-cell anemia (40–43).

## Conclusions

In summary, in population of ICU with stroke, we observed RDW levels were significantly associated with a poor prognosis in a positive linear fashion. Most importantly, we found that this association could be modified by AMI and HP. Our findings, if further confirmed, suggested that maintaining a lower level of myocardial injury markers, especially among patients with stroke or AMI, may offer a safer intervention strategy to reduce the mortality of stroke in ICU.

## Data Availability Statement

The data analyzed in this study was obtained from the eICU Collaborative Research Database, the following licenses/restrictions apply: Researchers seeking to use the database must formally request access. Requests to access these datasets should be directed to https://eicu-crd.mit.edu/gettingstarted/access/.

## Ethics Statement

The studies involving human participants were reviewed and approved by Massachusetts Institute of Technology (Cambridge, MA, USA). Written informed consent for participation was not required for this study in accordance with the national legislation and the institutional requirements.

## Author Contributions

TG contributed to conception and design of the study, drafted the manuscript, and performed the statistical analysis. ZQ organized the database. TG and DH reviewed the manuscript. All authors contributed to manuscript revision, read, and approved the submitted version.

## Conflict of Interest

The authors declare that the research was conducted in the absence of any commercial or financial relationships that could be construed as a potential conflict of interest.

## Publisher's Note

All claims expressed in this article are solely those of the authors and do not necessarily represent those of their affiliated organizations, or those of the publisher, the editors and the reviewers. Any product that may be evaluated in this article, or claim that may be made by its manufacturer, is not guaranteed or endorsed by the publisher.
