# Supplementary material for: Acute Myocardial Infarction (AMI) as the Effect Modifiers to Modify the Association Between Red Blood Cell Distribution Width (RDW) and Mortality in Critically Ill Patients With Stroke
Source: Front Med (Lausanne). 2022 Apr 26;9:754979. doi: 10.3389/fmed.2022.754979 (PMC9086673; doi:10.3389/fmed.2022.754979)
Supplement: Supplementary file 1 [file Table_1.doc]

Supplementary table 1: Subgroup analysis by stratified binary logistic regression model.

| Parameters | OR | 95%CI Low | 95%CI High | P-value | P(interaction) |
| --- | --- | --- | --- | --- | --- |
| GCS group |  |  |  |  | 0.0806 |
| 15 | 1.12 | 1.04 | 1.22 | 0.0053 |  |
| 13-14 | 1.05 | 0.95 | 1.15 | 0.3396 |  |
| 9-12 | 1.12 | 1.04 | 1.22 | 0.0040 |  |
| 3-8 | 1.01 | 0.95 | 1.07 | 0.8402 |  |
| Gender |  |  |  |  | 0.6901 |
| Male | 1.06 | 1.11 | 1.12 | 0.0447 |  |
| Female | 1.07 | 1.02 | 1.13 | 0.0073 |  |
| Ethniciy |  |  |  |  | 0.4043 |
| African American | 1.00 | 0.91 | 1.09 | 0.9444 |  |
| Asian | 1.14 | 0.86 | 1.51 | 0.3624 |  |
| Caucasian | 1.09 | 1.05 | 1.14 | <0.0001 |  |
| Hispanic | 0.91 | 0.67 | 1.23 | 0.5329 |  |
| Native American | 1.00 | 0 | inf. | 1.0000 |  |
| Unknown | 1.0 | 0.81 | 1.24 | 0.9883 |  |
| Acute respiratory failure |  |  |  |  | 0.0552 |
| No | 1.08 | 1.03 | 1.13 | 0.0007 |  |
| Yes | 1.00 | 0.93 | 1.07 | 0.9915 |  |
| Coagulopathy |  |  |  |  | 0.6041 |
| No | 1.07 | 1.03 | 1.11 | 0.0009 |  |
| Yes | 1.01 | 0.81 | 1.25 | 0.9382 |  |
| Diabetes mellitus |  |  |  |  | 0.1406 |
| No | 1.08 | 1.03 | 1.12 | 0.0004 |  |
| Yes | 0.99 | 0.88 | 1.10 | 0.9063 |  |
| Sepsis |  |  |  |  | 0.9817 |
| No | 1.07 | 1.02 | 1.11 | 0.0018 |  |
| Yes | 1.07 | 0.97 | 1.18 | 0.1935 |  |
| Stroke type |  |  |  |  | 0.1527 |
| Hemorrhagic stroke | 1.07 | 01.01 | 1.12 | 0.0206 |  |
| Ischemic stroke | 1.06 | 1.00 | 1.11 | 0.0426 |  |
| Others | 1.45 | 1.03 | 2.03 | 0.0328 |  |
| Cancer |  |  |  |  | 0.1596 |
| No | 1.06 | 1.02 | 1.10 | 0.0022 |  |
| Yes | 1.29 | 0.97 | 1.71 | 0.0749 |  |
| Age |  |  |  |  | 0.1637 |
| ＜65 | 1.03 | 0.96 | 1.10 | 0.4130 |  |
| ≥65 | 1.09 | 1.04 | 1.14 | 0.0005 |  |
